# Using the double diamond framework to co‐create and evaluate ‘1TeamActive’: A physical activity and well‐being intervention for police workers and their families

**DOI:** 10.1111/aphw.70197

**Published:** 2026-07-31

**Authors:** Owen Thomas, Helen Oliver, Tjerk Moll, Katherine Willoughby, Catharine Moss

**Affiliations:** ^1^ Cardiff School of Sport and Health Sciences Cardiff Metropolitan University Cardiff UK; ^2^ TeamPolice Tewkesbury UK

**Keywords:** family, physical activity, police, psychological well‐being, work stress

## Abstract

Police work can be stressful, negatively affecting well‐being and familial relationships. But, the influence of family‐based interventions on such outcomes is unknown. Using co‐creation, we employed the ‘Double Diamond’ framework in four British police forces to develop, deliver and evaluate ‘1TeamActive’ – a physical activity intervention for police workers and their families. Multiple stakeholders (Team Police UK, Sport England, four UK police forces, police workers and families) participated in four longitudinal research phases. In Phase 1, literature reviews ‘Discovered’ the stress impacts of policing (workers and families) and explored physical activity as a potential mitigator. In Phase 2, stakeholders ‘Defined’ an initial intervention plan, which was ‘Developed’ in Phase 3 to produce context‐specific delivery. In Phase 4, a 12‐week instructor‐led physical activity intervention was ‘Delivered’ and evaluated. One hundred and two police workers and their families completed the intervention. Repeated Measures Multivariate Analysis of Variance (RM‐MANOVA) indicated that physical activity, well‐being and work‐related variables improved significantly from pre‐ to post‐intervention, Pillai's trace = .65, *F*(8, 59) = 13.71, *p* < .001. Evaluation interviews indicated participants' perceived positive individual and familial benefits from completing ‘1TeamActive’. Our findings provide novelty and rigour with respect to intervention development, and the feasibility and acceptability findings support the intervention's potential to improve selected outcomes. Controlled trials are needed to determine efficacy.

## INTRODUCTION

Working in UK policing can be stressful, negatively impacting worker mental health, physical health and well‐being (Clements et al., 2021; Houdmont & Elliott‐Davies, 2016; Phythian et al., 2022). Police workers are exposed to operational stressors (e.g., witnessing traumatic events, experiencing violence) and organisational stressors (e.g., excessive workloads, shift work, lack of support from senior management), elevating the risk for mental ill‐health (e.g., depression, anxiety) and impaired physical health (e.g., cardiovascular disease, sleeping disorders; Clements et al., 2021; Gavin & Porter, 2024). Well‐being encompasses the absence of mental ill‐health outcomes as well as positive human functioning (e.g., positive relations with others, personal growth; Zhang & Chen, 2019), all of which are negatively impacted by police work (Sharp et al., 2022).

In response to the need to protect, promote and support police worker well‐being, the UK Home Office has launched the following: Oscar Kilo and the Blue Light Framework (Hesketh & Williams, 2017), the Front Line Review (Home Office, 2019), the National Police Well‐being Service and the Police Covenant (Home Office, 2022). The latter enshrined in law the requirement for UK government to report annually on police well‐being and support issues, improve the working experiences of those in policing, smooth the transition out of policing for workforce leavers and provide support for the families of those working in policing. To fulfil the Police Covenant (Home Office, 2022), evidence‐based approaches to supporting well‐being are needed (Phythian et al., 2022). Such approaches should evaluate the impact of police well‐being resources, in particular those which target individual‐based well‐being (e.g., promote healthy lifestyle, physical and social activity), as these interventions are less developed than practices focussed on organisational well‐being (e.g., absence management) in UK policing (Phythian et al., 2021, 2022). Existing individual‐based police well‐being interventions, which seem promising, include physical activity (Acquadro Maran et al., 2018), coping skills and wellness (Anshel et al., 2013). Yet, because of insufficient intervention reporting and/or small sample sizes, these interventions have not been replicated in more than one police force at a time. Further research is required to determine whether individual‐based interventions can be effective for well‐being *across* police forces to fulfil the Police Covenant (Home Office, 2022).

In the wider workplace literature (i.e., not solely focussed on police populations), physical activity is recognised as beneficial for worker well‐being because it enables psychological detachment from work stresses and promotes engagement with rewarding and challenging activities that produce positive feelings (Wiese et al., 2018). Despite these known benefits, day‐specific job demands have been shown to negatively influence workers motivation to undertake leisure‐time physical activity in several workplace communities (e.g., public/private sector administrative jobs; see Abdel Hadi et al., 2025, 2022). There is potential for such influences to be amplified in police workers because of the unique job demands and pressures they face (e.g., working long hours, shift work, lack of family time, trauma; see Gavin & Porter, 2024; Houdmont & Elliott‐Davies, 2016). Especially when some police‐specific research has shown that police workers are less likely to engage in physical activity after a stressful day than those in other occupational groups, despite perceiving that doing so would be beneficial to reduce stress (Sonnentag & Jelden, 2009). Rigorously reported research that accounts for the unique challenges of policing is needed to evidence if physical activity interventions are effective for improving police worker well‐being (see Oliver et al., 2024; Oliver, Thomas, Copeland, et al., 2022; Oliver, Thomas, Neil, et al., 2022).

Collaborative research is increasingly being used to improve the design, development and evaluation of interventions so they meet the needs of the individuals and groups receiving them (see Smith et al., 2022). Co‐creation is one such collaborative approach where participants (e.g., stakeholders, end‐users) partner in the research process (Vargas et al., 2022). Authors working in policing have recently integrated the Double Diamond framework (Design Council, 2019) into the co‐creation process to guide how researchers work with end‐users to understand and answer a problem (Oliver et al., 2024). Using co‐creation over a four‐year longitudinal period, our study aimed to employ the DD to ‘Discover’, ‘Design’, ‘Develop’, ‘Deliver’ and evaluate a family‐based physical activity intervention, which promoted the well‐being of police workers and their families in four British police forces. Our study was conducted in collaboration with *Team Police UK*, *Sport England*, and *four UK police forces*. By doing so, we provided a novel co‐creation intervention framework that stretched across multiple participating police forces, with a focus on intervention design and delivery that went beyond just police workers to include family groups—something clearly aligned with the Police Covenant (Home Office, 2022)—but not yet explored within the extant literature. We set three objectives to achieve this aim:

1. develop and deliver an intervention that could be implemented across a range of UK police forces;
2. evaluate any changes on physical activity, well‐being and work productivity following the intervention; and
3. evaluate perceptions of feasibility and acceptability of the intervention and perceived impacts on family relationships.

## METHOD

### Research design

**FIGURE 1:**
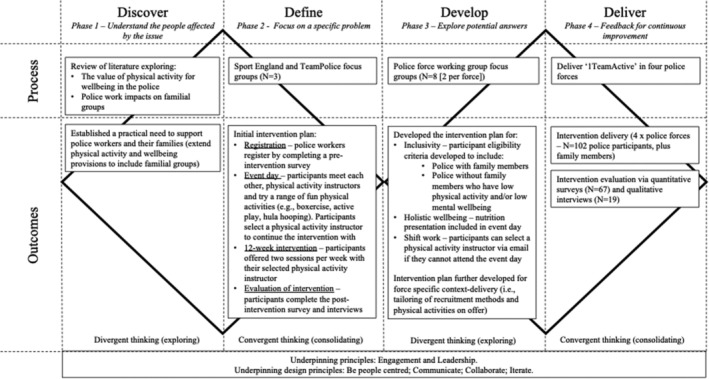
Intervention development process and outcomes, guided by Double Diamond (Design Council, 2019).

We adopted the DD ‘Discover’, ‘Define’, ‘Develop’, and ‘Deliver’ model (see Figure 1 and Data S1: Sport England evaluation framework). A multi‐method approach was adopted across the four DD phases. A range of quantitative (e.g., survey's, validated psychometric measures) and qualitative (e.g., focus groups and interviews) methods were used prior to a 12‐week delivery and evaluation of the intervention. In total, the study was conducted longitudinally over 4 years (2020–2023).

### Participants

**FIGURE 2:**
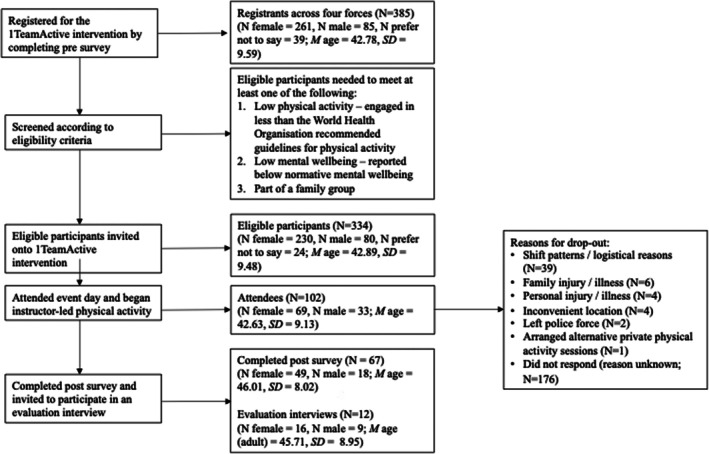
Participant flow.

Multiple levels of stakeholders participated across the longitudinal DD and intervention phases (see Figure 1: Intervention development process and outcomes guided by the Double Diamond framework):

- Sport England: a UK government funded body that aims to grow and develop grassroots sport and physical activity (Sport England, 2009). Sport England funded the research under the Families Fund (URN 2020021411) and participated as a stakeholder in Phase 2;
- Team Police UK: a subsidiary of Police Sport UK. Team Police UK provides opportunities and funds to support well‐being for those who have served in UK policing. Two senior Team Police UK representatives participated in Phases 2–4;
- Four UK police forces participated in the study. A working group was formed in each force led by their well‐being/wellness manager and supported by their teams. Police working groups participated in Phases 3 and 4.
- Intervention participants: 102 police participants were recruited across the four police forces to participate in Phase 4 with their families (see Figure 2: Participant flow).

To avoid potential concerns regarding bias or role overlap, no individual involved in the intervention development, design or delivery phases was a direct participant (recipient) in the intervention phase.

### Procedure

Ethical clearance from the lead author's institution was granted (reference: Sta‐4138). To support methodological rigour, as recommended in the CONSORT (Consolidated Standards of Reporting Trials) statement (Hopewell et al., 2025), the Template for Intervention Description and Replication (TIDieR) checklist (Hoffmann et al., 2014) was used to ‘Define’ and ‘Develop’ the intervention (see Data S2: TIDieR checklist).

#### Phase 1 (Discover)

Literature reviews were conducted to ‘Discover’ whether physical activity is an appropriate intervention method to support police worker well‐being and how police work affects familial groups. An electronic search strategy was conducted on six databases (PscyINFO, PsycArticles, EMBASE, MEDLINE, SportDiscus and Scopus), using the keywords *police, work stress, well‐being, physical activity* and *family*.

#### Phase 2 (Define)

The ‘Define’ phase involved a series of focus groups (*N* = 3) with Sport England and Team Police, UK representatives that were used to ‘Define’ an initial intervention plan based on Phase 1 findings.

Informed by the Sport England evaluation framework (see Data S1: Sport England evaluation framework), these focus groups defined the project (Focus Group 1); set evaluation goals and agreed the scope of the intervention (Focus Group 2); and planned data collection (Focus Group 3). Focus groups lasted 60–75 min and were transcribed verbatim yielding 45–68 pages of transcribed data.

#### Phase 3 (Develop)

In the ‘Develop’ phase, two focus groups were conducted with each police force working group (total *N* = 8[2 × 4]), tailoring intervention delivery to each force context. In Focus Group 1, forces gave feedback on the initial intervention plan and produced a refined plan. In Focus Group 2, forces tailored the refined plan so that it was deliverable in their context. Focus Group 1 lasted between 90 and 110 min, whereas Focus Group 2 lasted between 60 and 85 min. Both sessions were transcribed verbatim, yielding 194 pages of transcribed data.

#### Phase 4 (Deliver)

In Phase 4, the intervention was ‘Delivered’ and evaluated across the four police forces using a single‐group pre–post design. Participant eligibility criteria were developed with police force working groups in Phase 3 (Results). Criterion‐based and key informant sampling via Occupational Health Services were used to recruit police force workers. Opportunity sampling via poster recruitment was also used (see Data S2: TIDieR checklist). The intervention was ‘Delivered’ in each police force over a 12‐week period. To evaluate the intervention, the participants completed a pre‐and‐post‐intervention quantitative psychometric survey (i.e., International Physical Activity Questionnaire – Short Form; IPAQ‐SF [Booth, 2000]; Warwick‐Edinburgh Mental Well‐being Scale; WEMBS [Tennant et al., 2007]; The Psychological Need Satisfaction in Exercise Scale – PNSES [Wilson et al., 2006]; Work Limitations Questionnaire – WLQ [Lerner et al., 2001]; and specific items from the Sport England Active Lives Survey [Sport England, 2025] – see Data S2, TIDieR checklist). A subset of participants from each force completed a semi‐structured evaluation interview (*N* = 12 interviews). Interviews lasted between 27 and 38 min and were transcribed verbatim yielding 111 pages of transcribed data. Quantitative data were analysed via Repeated Measures Multivariate Analysis of Variance (RM‐MANOVA) and qualitative data via reflexive thematic analysis (Braun & Clarke, 2022).

### Materials

Police force working groups received three support packages to guide intervention delivery within their force. Support packages included all the information needed to recruit participants, collect pre‐and‐post intervention quantitative psychometric data and plan for the 1TeamActive intervention. Data S2 (TIDieR checklist) details all the materials provided in the support packages. A semi‐structured interview schedule was also developed for data collection (see Data S3: 1TeamActive interview schedule).

## RESULTS

### Phase 1 (Discover)

Our review of literature (Oliver, 2022; Oliver et al., 2023) and previous empirical research (Oliver et al., 2024; Oliver, Thomas, Copeland, et al., 2022; Oliver, Thomas, Neil, et al., 2022) found that physical activity can reduce perceptions of stress and improve well‐being in police workers. Police officers with low physical activity behaviour had significantly lower well‐being than those who engage with physical activity (Bradley et al., 2023), while police officers with increased fitness perceived less stress (Gerber et al., 2010). However, there were few examples of police physical activity interventions that have been informed by theory (Oliver et al., 2023). Suggested theoretical mechanisms to explain the potential benefits of physical activity and to underpin intervention effects tended to draw upon Self‐Determination Theory (SDT; Ryan & Deci, 2000), which postulates that individuals' experience well‐being when three basic psychological needs (relatedness, competence and autonomy) are met. Some research within police populations has suggested that context and the undertaking of physical activity might support well‐being via fulfilling feelings of self‐determination through the satisfaction of basic needs, but specific intervention research is required (Oliver et al., 2024). ‘Relatedness’ was considered particularly salient because of our focus on including family groups and the potential to influence meaningful family/co‐worker relationships.

Our review also found that the impact of police workplace stress was prominent on families (McQuerrey Tuttle et al., 2018; Sharp et al., 2022). The term ‘spillover’ effects described how stress‐related cognitions, affects and behaviours transfer from the occupational domain or ‘spillover’ into non‐work family settings (Burke, 1993; McQuerrey Tuttle et al., 2018). Such impacts were suggested to affect marital and dyadic relationships and broader family unit functioning (Brodie & Eppler, 2012). Context‐specific police research indicated that missing family activities, choosing work over family, scheduling and shift conflicts, career demands and emotional spillover of work‐related stress into homelife were stress inducing consequences for marital and family relationships (Karaffa et al., 2015; McQuerrey Tuttle et al., 2018; Papazoglou & Tuttle, 2018). However, intervention research exploring strategies to mitigate such stress effects was scarce, tending to focus solely on officer's responses or coping (McCraty & Atkinson, 2012), thus overlooking dyadic/familial spillover effects (McQuerrey Tuttle et al., 2018). Alongside the principles of the Police Covenant (Home Office, 2022), we deemed this a critical omission given the known deleterious effects workplace stress can pose to marital and family subculture (Karaffa et al., 2015; Sharp et al., 2022), workplace performance and well‐being (Gavin & Porter, 2024). Existing police worker wellness intervention research has positively influenced officer perceptions of distress, anger, sadness and fatigue, and improved communication (McCraty & Atkinson, 2012). Although researchers have suggested such effects *could* translate into family support interventions, with beneficial consequences for dyadic and familial relationships (e.g., influencing family coping, well‐being perceptions and communication; McQuerrey Tuttle et al., 2018), such proposals remain hypothetical and are not yet empirically evidenced (McQuerrey Tuttle et al., 2018; Papazoglou & Tuttle, 2018).

### Phase 2 (Define)

The first focus group reviewed Phase 1 findings. Our Phase 1 research reinforced physical activity as an efficacious method to improve psychological well‐being for those working within UK policing (Buckingham et al., 2020; Gerber et al., 2010; Poirier et al., 2023) and indicated potential extension of these benefits to marital and family groups (Karaffa et al., 2015; Sharp et al., 2022). Therefore, the first focus group defined that 1TeamActive would focus on improving well‐being for police workers and their families through the target behaviour of physical activity. Further, Phase 1 findings also pointed to adopting SDT as a broad underpinning theoretical framework for our intervention. This view was shared by participants in the first focus group and aligns with Medical Research Council (MRC) guidance, which recommends that complex health interventions be grounded in a clearly articulated theoretical framework (Skivington et al., 2021).

In the second focus group, the scope and goals of the 1TeamActive evaluation were set. Using the extant literature on well‐being and physical activity interventions in the police (Buckingham et al., 2020), the Sport England evaluation framework (see Data S1: Sport England evaluation framework) and discussion in the focus group, quantitative psychometric survey measures for use before and after participation in 1TeamActive were agreed and selected. Physical activity was measured with the IPAQ‐SF (Booth, 2000). In line with our earlier definition of well‐being, mental health outcomes (presence or absence of) were measured by the WEMBS (Tennant et al., 2007); and positive human functioning was measured via a proxy measure of self‐determination through the satisfaction of basic needs in physical activity/exercise contexts (PNSES; Wilson et al., 2006). Work productivity was measured using the Work Limitations Questionnaire (WLQ; Lerner et al., 2001). Additional specific items from the Sport England Active Lives Survey (goals, anxiety) were also included (Sport England, 2025; see Data S2: TIDieR checklist). To gather perceptions of the impacts of 1TeamActive for family members, it was agreed that qualitative evaluation interviews would be used.

In the third focus group, the initial 1TeamActive intervention plan was ‘Defined’ (see Figure 1: Intervention development process and outcomes guided by the Double Diamond framework). The plan specified that participants and their families would be invited to a 1TeamActive event day (see Data S4: 1TeamActive event day schedule) where they would meet other participants and physical activity instructors, and try a range of fun physical activities (e.g., boxercise, active play, hula hooping) to engage with a 12‐week intervention period. In line with SDT and basic psychological needs frameworks, attending the event day in dyadic/family groups was important to build participants' competence (i.e., realise their capability of engaging in exercise/physical activity), autonomy (i.e., intervention physical activity choice) and relatedness (i.e., connectedness as a dyad/family and/or with other participants; Quested et al., 2021).

### Phase 3 (Develop)

#### Police force working group focus groups

The first focus group with police force working groups discussed their feedback on 1TeamActive. Working groups were supportive and enthusiastic about the intervention once initial concerns about the workload involved with facilitating the intervention were alleviated. For inclusivity, the intervention plan was refined so that police workers without family members as well as police workers with family members could apply to participate in 1TeamActive. Further, as Phase 1 found psychological well‐being benefits of physical activity were most significant for police workers who were less active or suffering with mental ill‐health (Buckingham et al., 2020; Oliver, Thomas, Neil, et al., 2022), the refined plan specified that individual police workers could apply to participate in 1TeamActive if they met this criteria (see eligibility criteria in Figure 2: Participant flow). Police force working groups also suggested including a nutrition presentation within the 1TeamActive event day, so holistic well‐being was supported. To address concerns that police shift patterns may prevent participants attending the event day, the intervention was also refined so that participants could work with physical activity instructors without meeting them at the event day (see Figure 1: Intervention development and process and outcomes guided by the Double Diamond framework).

In the second focus group, adaptations to deliver 1TeamActive in the four police force contexts were discussed. Contextual differences included the methods to recruit participants and the physical activities offered (see intervention tailoring in Data S2: TIDieR checklist). As Sport England Active Partnerships were utilised to access physical activity instructors in each police force region, different physical activities were available (TeamPolice, 2023).

### Phase 4 (Deliver)

**FIGURE 3:**
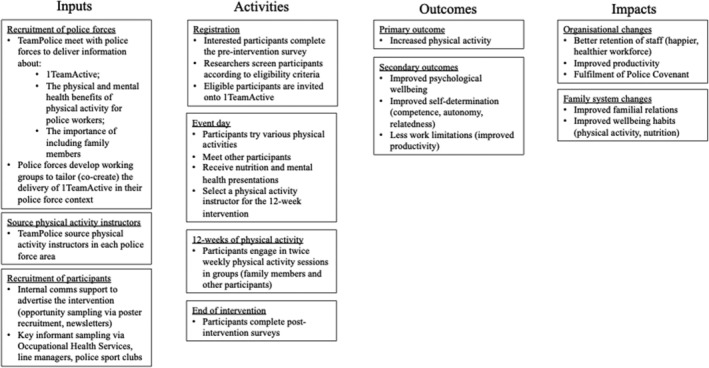
Logic model (programme theory).

The final intervention is summarised in Figure 3 (Logic model for 1TeamActive), with additional detail in Figure 2 (Participant flow) and the ‘Develop’ and ‘Deliver’ phases of Figure 1. In short, intervention delivery comprised an event day during which participants got to know each other, trialled various physical activities and received well‐being presentations (i.e., on nutrition and mental health; see Data S4: 1TeamActive event day schedule). After the event day, the participants and their families selected a physical activity for the 12‐week intervention period (Team Police, 2023).

#### Quantitative results: changes in physical activity, well‐being and work‐related variables

Of the initial 102 participants, a total of 67 completed both pre‐ and post‐intervention surveys. The participants who failed to complete all post‐surveys (*n* = 33) or all pre‐surveys (*n* = 2) were excluded. Of the 67 participants, 59 participants completed all measures within the pre‐ and post‐surveys, five participants missed one measure within the post‐survey phase, two participants missed one measure within the pre‐survey phase and one participant missed three measures within the post‐survey phase (see Data S5). Missing data for all cases were treated using mean imputation (Van der Heijden et al., 2006). A sensitivity power analysis (G‐power version 3.1; Faul et al., 2007) was conducted to determine what effect size could be detected with the sample size of 67 for a within‐subjects repeated measures with time (pre‐ and post‐intervention) as the within‐subjects factor (Lakens, 2022). With power set at 0.80, an alpha of 0.05 and a correlation of 0.5 among the repeated measures to detect a significant effect of time, the sample was powered to detect small‐to‐medium effects (*f* = 0.17, Pillai's trace = 0.11).

Prior to the main analyses, descriptive statistics were calculated (see Table 1). Also, one‐way between groups multivariate analysis of variance (MANOVAs) revealed no potential confounding effect of the demographic variables, gender (Pillai's trace = 0.23, *F*[16,50] = 0.93, *p* = .55) and age (Pillai's trace = 0.22, *F*[16, 48] = 0.84, *p* = .64) on the outcome measures. Hence, these variables were not controlled for in subsequent analyses. The main Repeated Measures (RM)‐MANOVAs indicated significant changes in physical activity, well‐being and work‐related variables pre‐to‐post‐intervention, Pillai's trace = 0.65, *F*(8, 59) = 13.71, *p* < .001. When the dependent variables were considered separately, using a Bonferroni adjusted alpha for multiple comparisons, significant changes were observed. There were significant effects for physical activity (*F*[1, 66] = 39.73, *p* < .001, *ŋ* ^2^_*p*_ = 0.38); competence (*F*[1, 66] = 8.88, *p* < .01, *ŋ* ^2^_*p*_ = 0.12); relatedness (*F*[1, 66] = 5.02, *p* < .05, *ŋ* ^2^_*p*_ = 0.07); well‐being (*F*[1, 66] = 84.65, *p* < .001, *ŋ* ^2^_*p*_ = 0.56); and work limitations (*F*[1, 66] = 5.44, *p* < .05, *ŋ* ^2^_*p*_ = 0.08). Specifically, post‐intervention, the participants were significantly more physically active and competent about engaging in exercise/physical activity; reported more relatedness and better well‐being; and experienced fewer work limitations than pre‐intervention (see Table 1). No significant pre‐to‐post‐intervention differences were found for autonomy, anxiety or ability to achieve goals (self‐efficacy).

**TABLE 1:** Descriptive statistics and univariate effects of RM‐MANOVA for physical activity, well‐being and work‐related variables.

Outcome variable	Time‐point	Min	Max	*Z*_Skewness_	*Z*_Kurtosis_	*M* (*SD*)	*F*	*ŋ* ^2^_*p*_
Physical activity (MET minutes)	Pre	0	6745	7.71	15.52	1128.01 (1183.71)	39.73**	0.38
	Post	0	10,662	4.83	2.95	2831.52 (2505.06)		
Competence	Pre	1	6	1.26	0.29	3.24 (1.15)	8.88*	0.12
	Post	1	6	1.21	1.32	3.81 (1.35)		
Autonomy	Pre	1	6	0.98	0.88	4.14 (1.29)	3.39	0.05
	Post	1	6	2.48	0.98	4.50 (1.17)		
Relatedness	Pre	1	6	0.05	1.13	3.37 (1.36)	5.02*	0.07
	Post	1	6	1.75	0.82	3.91 (1.25)		
Well‐being	Pre	3	30	0.51	1.80	16.45 (6.65)	84.65**	0.56
	Post	12	35	1.74	2.32	25.02 (4.14)		
Anxiety	Pre	0	10	0.21	1.03	4.28 (2.97)	2.11	0.03
	Post	0	10	2.43	1.74	3.76 (2.91)		
Goals	Pre	1	5	1.33	1.68	3.22 (1.09)	1.99	0.03
	Post	1	5	1.41	1.15	3.41 (1.04)		
Work limitations	Pre	18.75	81.25	3.25	1.01	39.59 (14.05)	5.44*	0.08
	Post	12.50	68.75	2.13	0.49	35.86 (12.82)		

Abbreviation: RM‐MANOVA, Repeated Measures Multivariate Analysis of Variance.

* *p* < .05,

** *p* < .01.

#### Qualitative results: evaluation interviews

Twelve interviews were conducted with 24 participants (seven were individual interviews, five were with dyad/family groups). In total, the interviews comprised 12 police participants, five spouses and eight children. There were nine males and 16 females. Three themes were identified across the 12 interviews. ‘*Individual impact*’ had three subthemes: *Psychological, Physical* and *Social*. The second theme, ‘*Family impac*t’ had two subthemes: *Relationships* and *Habits*. The third subtheme was ‘*Feasibility and acceptability*’.

The participants suggested that the 1TeamActive intervention had a *positive psychological* impact on them as individuals. For example, one participant explained:

When you exercise, it does actually make you feel better. I know that it makes you feel better physically and emotionally, because when you have finished you are like, ‘oh, I'm so tired’, but it feels really good that you have performed something. Because it is a challenge for me and it is difficult for me, so when I finish, it I feel like I am proud of myself.

Other participants said that physical activity had been a ‘*stress relief’*, ‘*helped them to relax’* or that they ‘*came out of it* [physical activity] *feeling a lot more refreshed and energized’*. The participants also felt improvements in their physical well‐being following the intervention:

*… from week one to week 12 the difference* [in fitness] *was amazing. On week one when we did a plank, I managed nine seconds, and on week ten I think I managed about 48 seconds. You could see the progress … And being able to get a coat done up at the end of it as well. It was a coat that I couldn't wear before. I was like: Oh, hello!*

Engaging in the 1TeamActive intervention also helped participants feel more confident in *physical activity*:

*I do feel fitter* – *one of my goals was possibly to drop just a few pounds because I am not overweight by any stretch … I am still working on that aspect* – *but I do feel fitter and I was surprised how much stamina I have now got actually. I couldn't run 10 kilometers, I didn't think I ever could do really, but now I think actually if I tried I could. It's one of those things where you have just got to go for it and see what happens! I think I have gained the confidence to try it and keep going as well. So, that has been really good. I think that is the main thing, is feeling I can actually do something, where it is building up your strength or whatever, and you can actually see the effect happening over weeks and weeks*.

Several *positive social impacts* were also found from participating in 1TeamActive:

*I really enjoyed it* [1TeamActive]*. It was because it was a team, you felt you were letting the team down, if you didn't go and then, when you're there, you're competing with and against each other but in a fun way. So yeah, there was a bit of rivalry between the team*.

The *positive social impacts* also benefitted work relationships:

*I knew* [supervisor at work] *already because she was my boss actually. And, her and I usually partnered up and she'd say nice things like ‘oh you are a good partner to work with’, she is very good at spurring people on which is why she's the boss! I can see why she is in the position she is in at work because she is always giving you little pointers and stuff like that. So it was nice to get that connection outside of work and then take that back into work too*.

1TeamActive provided *positive family impact*, benefitting family relationships by providing quality time for the family to do something:

*DAD*
*Well, after* [1TeamActive ended] *we were bored*

*MUM*
*We were really bored, yeah*

*DAD*
*For like three, four days a week we were going out to do stuff in the evenings* [with 1TeamActive] *but then we just stopped and were: Oh, it's a bit pants now isn't it?*

*CHILD 2*
*And it made me feel more social because we spent more time with our family, like quality time*

*MUM*
*Yeah. It was more of a purpose, so we'd come home, do the school run, cook tea, come in, get changed, go, and then sort of do an hour* [of 1TeamActive physical activity]*, come back, and then… Yeah, it was a bit more structured than… Now the evenings sort of…*

*CHILD 1*
*Roll all together*

*MUM*
*Yeah, they do roll a bit, really*.

The participants also stated how the 1TeamActive intervention was effective in helping them improve various *family habits*. One participant spoke about how the nutritional information in the 1TeamActive intervention had a positive impact on her family:

*I thought we ate healthy foods but for me personally, I could go out the door having had no breakfast, skip lunch because I'm busy at work, and until we started doing this* [1TeamActive]*, I didn't realize I literally survived on cups of tea throughout the day until teatime, which was my first meal of the day several times a week. And, I didn't even realize I was doing that…. I was running on empty. So, the whole family eat breakfast now. You must eat your breakfast. You must put fuel in. And, just that little one change … it's positive impacts and it's quite shocking the amount it impacted me from such a small change. I totally know you can fit it in your day just, having a bit of breakfast or, going for a walk round the block on the night before, just those little things, it's not even necessarily just for your health; it just makes you feel more positive*.

Another participant spoke about how their Sunday routine had changed following the intervention, to include more physical activity:

*The* [1TeamActive] *course gave us that incentive to try and do things as a family. So we brought Sunday dinner forward to Sunday lunch and then went for a family walk afterwards*.

The participants agreed that 1TeamActive was a *feasible and acceptable* physical activity intervention for police force workers and their families. One participant explained why the 1TeamActive intervention was particularly needed for police force workers:

*Every single police force has units staffed by sedentary people, whether that's the admin staff or the IT department or the forensics department. Obviously, the cops tend to be a lot more active, because they are out there, they have to stay a certain level of physical fitness and pass fitness tests. But there are huge waves of police staff who none of that applies to. So, there would be a good proportion* [of police] *who absolutely do need that extra push to get fit, and working for a police force can be very stressful at times, so if the police are going to offer schemes like this to help improve people's mental health then I think it should be rolled out to every force in the country*.

All participants indicated that they would like to sign up to the 1TeamActive intervention again; however, there was a variation in whether participants maintained their physical activity themselves after the intervention had finished. Some continued as families and in their groups *“We've kept it going ‐ we're paying for it* [physical activity sessions] *now, but there was enough of us in our group to warrant him* [the instructor] *doing a class, so it's us continuing to do it”*. However, not all participants could afford to pay for their sessions once the intervention ended or had lower numbers in their activity groups, which limited their perceived ability to continue being active once the 1TeamActive intervention ended.

The participants also mentioned that there were barriers for shift workers in attending the intervention: ‘*… because of working shifts, it was difficult to get to some of the sessions during the 12 weeks and I didn't actually make all the sessions*.’ The participants felt that, with organisational support, shift workers could be reached and the intervention could run sustainably within their police force:

.*..they* [shift workers] *should be supported by a line manager. So, if the session's at 18.15 you should be allowed to knock off at half four to go home and get there. So it might be that if you've got a police sponsor, he could also potentially check in with my sergeant to say there is flexibility available. Because I think that will be an excuse you find a lot and could probably combat that if you've got that internal contact*.

## DISCUSSION

Responding to UK policing policy (Home Office, 2022), extant literature surrounding stress and well‐being in police workers (Phythian et al., 2021, 2022), and spillover effects to their families (Karaffa et al., 2015; McQuerrey Tuttle et al., 2018; Papazoglou & Tuttle, 2018), our research used co‐creation over a longitudinal period to develop, deliver and evaluate a physical activity intervention with police force workers and their families. We found that the DD and Sport England evaluation frameworks supported the engagement of multiple stakeholders in the 1TeamActive physical activity intervention in four UK police forces (objective 1). Phase 1 (‘Discover’) found police work ‘spills‐over’ to negatively influence family well‐being; and that in line with SDT, physical activity can support police well‐being. In Phase 2 (‘Define’) and Phase 3 (‘Develop’), the 1TeamActive physical activity intervention was co‐created over a series of focus groups to produce a physical activity intervention (see logic model in Figure 3), which was tailored for contextually feasible delivery across four UK police forces (see Data S2: TIDieR checklist). In Phase 4 (‘Deliver’), the intervention was delivered using a single‐group pre‐post design over a 12‐week intervention period in each police force. Quantitative results demonstrated that compared to pre‐intervention, police participants were significantly more physically active, demonstrated improvements in positive human functioning by being significantly more competent about their perceptions of engaging in exercise/physical activity, felt significantly more related towards those they were undertaking exercise/physical activity with, experienced significantly better mental well‐being and reported significantly fewer work limitations following the intervention (objective 2). Qualitative evaluation interviews indicated that 1TeamActive was a feasible and acceptable intervention for implementation in police forces, provided that sufficient organisational support was available. Family members also reported positive impacts following 1TeamActive for themselves as individuals and for their familial relationships and well‐being habits (objective 3).

Our longitudinal study provides preliminary empirical evidence of the positive influence that context‐specific well‐being interventions can have on police workers *and* their families within the UK policing community. It also established the impact that novel co‐created physical activity interventions can have on the well‐being of police workers (Oliver et al., 2024). Police participants reported improved positive human functioning, mental well‐being and work productivity following the 1TeamActive intervention, answering calls surrounding the viability of physical activity as an effective intervention for workplace wellness in policing contexts (Acquadro Maran et al., 2018). Alongside considering the influence on police workers, we have answered legislative calls to consider the support received by the families of police workers (i.e., Police Covenant; Home Office, 2022). Our work therefore shows promise in addressing a gap in the existing evidence base (Phythian et al., 2022). Qualitative evaluation interviews indicated that family members (spouses and children) perceived improved relationships within their families after participating in 1TeamActive, providing an important insight into how the deleterious effects of police work‐related stress on the wider family context can be mitigated (McQuerrey Tuttle et al., 2018). We know policing can have negative impacts on worker well‐being, which ‘spillover’ to negatively affecting family members (e.g., McQuerrey Tuttle et al., 2018; Sharp et al., 2022); our new findings hold early promise for suggestions that family‐based intervention programmes (here via physical activity) can positively influence relationships within the family dyad. Exploring other intervention modes beyond physical activity would be useful. The interviews with police workers also emphasised how organisational support affected participant engagement in such physical activity–type interventions. Findings connect well with the broader recovery from work literature (e.g., Sonnentag et al., 2022) and more specifically with respect to day‐level effects of physical activity on employee well‐being (e.g., Abdel Hadi et al., 2022; Roswag et al., 2023).

At a conceptual level, our finding that competence (about engaging in exercise/physical activity) and relatedness (towards those they were undertaking exercise/physical activity with, that is, family member and work colleagues) significantly increased following the 1TeamActive intervention supports contentions that SDT and the satisfaction of basic needs can act as an underpinning mechanism by which physical activity can improve both positive human functioning and mental well‐being for police workers (Fortier et al., 2012; Teixeira et al., 2012). Other police research has used SDT as an underpinning framework for stress management‐related interventions (e.g., Jones et al., 2020) but not used physical activity as the target behaviour for well‐being improvements. Our research offers early evidence that such intervention modes are appropriate for this group and context when trying to target stress and well‐being, something until now only evidenced by research in contexts and/or groups outside of policing (e.g., Fortier et al., 2007; Quested et al., 2021).

There are also new methodological impacts from our work. Complex health interventions should evidence a clear process, have a theoretical basis and appreciate context (Skivington et al., 2021), something rarely achieved in research (Duncan et al., 2020). Our combined use of co‐creation and the integration of the DD framework provided a novel methodological process that included theory, stakeholder(s) and workforce engagement, evidencing clear appreciation of context (Oliver et al., 2024). This holistic approach shows promise, and other researchers can use this framework to co‐create other ecologically valid interventions that are feasible and appropriate for their context (Oliver et al., 2024). Our research process is translatable across settings as an approach, so others can use it to develop different evidence‐based, co‐created interventions.

### Strengths and limitations of this study

Our study has strong ecological validity and was undertaken in a novel context with new populations (i.e., police workers and their family dyads). We also used robust methods over several longitudinal phases in a theoretically driven approach. Our adoption of co‐creation and the DD alongside each other in one design was also novel. Individually, these elements are clear strengths, but holistically, and in combination, they evidence a rigorous approach to intervention research and align with the new Medical Research Council guidance for developing and evaluating complex health interventions (see Skivington et al., 2021). However, despite these design strengths and our finding that 1TeamActive was beneficial for the well‐being of police workers and their families, there are limitations to the work. For example, the final intervention phase (Phase 4 – Deliver) of our work used a single‐group pre–post intervention design and measures of self‐report to determine intervention effectiveness. And although, given our design, we make no claims regarding intervention efficacy, the use of a more complete experimental or quasi‐experimental design with control or comparison conditions and more objective measures of behaviour change (e.g., accelerometer or wearable activity tracking data) could have provided greater solidity around possible claims of intervention strength (Marsden & Torgerson, 2012). Additionally, although it is not uncommon for drop‐out rates in physical activity intervention research to range between 25% and 50% (e.g., Collins et al., 2022; Kraus et al., 2001; Linke et al., 2011). The drop‐out rate of eligible participants from registration to intervention was moderate (30%; see Figure 2: Participant flow). Despite their initial interest, logistical and shift pattern issues prevented participants' long‐term adherence to the intervention (see Figure 2: Participant flow). Such demands are not uncommon for police workers (e.g., Clements et al., 2021; Gavin & Porter, 2024), but the drop‐out rate potentially limits the generalisability and representativeness of our findings and increases the risk that our sample represented a motivated subset of the initial participant pool (Collins et al., 2022). Future research should explore how physical activity and/or well‐being interventions can be more easily accessed by police workers and their families to help better inform police well‐being policy (e.g., the Police Covenant; Home Office, 2022). Finally, one outcome of the co‐creation process was that the stakeholders wished the intervention to target participants with low physical activity levels and low well‐being, such eligibility criteria possibly limited the target population of the research and realised a sample which were mostly female.

## CONCLUSION

In this longitudinal study, co‐creation was used to iteratively co‐develop and deliver 1TeamActive, a physical activity intervention for police force workers and their families. Following completing the 1TeamActive intervention, physical activity levels, well‐being, aspects of motivation (competence and relatedness) and work productivity improvements were evidenced in workers from four British police forces. Police workers and their family members (spouses and children) also perceived positive psychological, physical and social impacts at an individual level and positive relationship impacts at a family level following the 1TeamActive intervention. Our research supports the idea that physical activity as a target behaviour can support police well‐being and that SDT and the satisfaction of basic needs realised through participation in physical activity/exercise can act as a broad theoretical framework to help account for such effects. Further, we provide novel contributions regarding the co‐creation process and intervention design. We also offer preliminary evidence‐based solutions for mitigating the negative ‘spillover’ stress effects police work can impart on familial relationships. Future research should continue to consider family members in police well‐being interventions using more complex intervention designs and engage in co‐creation processes to ensure that, like our research, interventions are theoretically underpinned, acceptable and feasible, and contextually rich.

## CONFLICT OF INTEREST STATEMENT

We declare no competing interests.

## ETHICS STATEMENT

All procedures within this study were performed in accordance with the ethical standards of the lead author's institutional ethics committee. Approval for the study was obtained from the Research Ethics Committee of Cardiff Metropolitan University (reference: Sta‐4138).

## Supporting information


**Data S1.** Sport England evaluation framework.

**Data S2.** TIDieR checklist.

**Data S3.** 1TeamActive interview schedule.

**Data S4.** Supporting Information.

**Data S5.** Specific missing measure/scale data points by participant.

## Data Availability

Open‐source quantitative data are available from 10.25401/cardiffmet.32974058. We do not have permission to share the raw qualitative interview transcripts because of the populations involved. We do have permission to share the quotes used within the paper. Please contact the corresponding author for the information.
