# Supplementary material for: Using the double diamond framework to co‐create and evaluate ‘1TeamActive’: A physical activity and well‐being intervention for police workers and their families
Source: Appl Psychol Health Well Being. 2026 Jul 31;18(4):e70197. doi: 10.1111/aphw.70197 (PMC13428052; doi:10.1111/aphw.70197)
Supplement: Supplementary file 1 — Data S1. Sport England evaluation framework. [file APHW-18-0-s003.pdf]

Evaluation Framework: The Essentials

Sport England's Evaluation Framework is a step-by-step guide to measuring and evaluating your project – but if you're new to evaluation, short on time, or just want a quick reference, this accessible summary is the place to start.

Good evaluation helps you improve your project, and prove how effective it is. This helps you provide a better service for the people using it, and attract more funding and support in the future. For Sport England it provides further evidence of the benefits of being active – including the Government's five Outcomes for sport – and the best way to achieve them.

The steps below take you through a project evaluation from start to finish. Begin Step 1 as soon as you start designing your project (or writing your funding application), so that evaluation is built into your project and runs alongside it.

TIP: How you complete each task is up to you and will depend on the time, resources and expertise you have available. For example, the first task in Step 1 might be a discussion, a meeting, a series of workshops or even a piece of research. Our advice is: **keep it proportionate, and make it work for you.**

Step	Description	✓ Essential tasks
1 **Define your project**	This is your foundation: describe your project, why it's needed, what you want to achieve, and how you will do it.	☐ Set out what you want to achieve (your project objectives)<br>☐ Agree what *changes* (outcomes) will lead to your objectives<br>☐ Agree what you will do or deliver to cause these changes<br>☐ Use evidence, insight and partner views to develop your project<br>☐ Confirm your final project design
2 **Set your evaluation goals**	Identify and prioritise the most important things you need to measure and evaluate, and what else you hope to learn.	☐ Set your evaluation objectives: what questions will it answer?<br>☐ Consider how (and with whom) you want to use the findings<br>☐ List the things you will measure that will indicate project success<br>☐ Identify anything else you hope to learn (learning goals)
3 **Agree the scope**	Decide on the size and scope of your evaluation, whether you need external support, and your available budget.	☐ Scope out an evaluation balanced to your resources and needs<br>☐ Decide whether to manage it yourself, or find external support*<br>*(*If you do, work with them on Steps 4-6, and revisit Steps 1-2)*<br>☐ Confirm your evaluation budget
4 **Plan your data collection**	Finalise precisely what data and information you need, and choose which methods, tools and systems you will use to collect it.	☐ Specify what you will collect to fulfil your evaluation objectives (aim for a mix of numerical data and descriptive information)<br>☐ Choose how, when and where you will collect it (your method)…<br>☐ …and select what 'tools' you will use, such as questionnaires<br>☐ Set up any software or systems you will use to store data<br>☐ Check your data collection and storage will comply with GDPR
5 **Collect the data**	Confirm the practical steps you will take to develop and distribute your tools; then collect, collate and analyse your data.	☐ Create, sign-off and issue your data collection tools from Step 4<br>☐ Confirm data collection arrangements with your staff<br>☐ Collect the data (using your method from Step 4)<br>☐ Gather or group your data as needed, e.g. onto a spreadsheet<br>☐ Analyse your data to see what it tells you
6 **Use the findings**	Use and share what you have learned, to improve delivery, show your impact and help your organisation and others.	☐ Use what you learn to adapt delivery and improve your project<br>☐ Review data regularly to track your project's progress<br>☐ Report essential data, e.g. to your funder or internally<br>☐ Tailor and share findings externally to show success and learning

Visit our full framework for more guidance, a glossary and resources including **Question Banks** for Adults and Children.
If you have comments, questions or feedback, please email us at se.evaluation@sportengland.org. Thank you! ☺
