# Supplementary material for: Using the double diamond framework to co‐create and evaluate ‘1TeamActive’: A physical activity and well‐being intervention for police workers and their families
Source: Appl Psychol Health Well Being. 2026 Jul 31;18(4):e70197. doi: 10.1111/aphw.70197 (PMC13428052; doi:10.1111/aphw.70197)
Supplement: Supplementary file 2 — Data S2. TIDieR checklist. [file APHW-18-0-s002.docx]

**Supplement File 2 – TIDieR checklist**

**
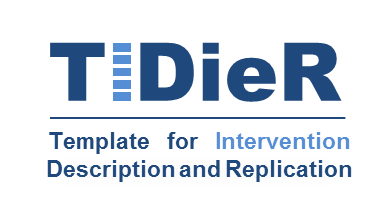
The TIDieR (Template for Intervention Description and Replication) Checklist*:**

Information to include when describing an intervention and the location of the information

| **Item number** | **Item** | **Where located **** | |
| --- | --- | --- | --- |
|  |  | Primary paper  (page or appendix  number) | Other ^†^ (details) |
|  | **BRIEF NAME** |  |  |
| **1.** | *The 1TeamActive physical activity and well-being intervention for police workers and their families* | ____________ | _____________ |
|  | **WHY** |  |  |
| **2.** | *Rationale and theory:*  *Police force workers have highly stressful, demanding and emotionally challenging roles which negatively impact their well-being and can ‘spillover’ to also negatively impact their dyadic, marital and familial relationships*(1)*. Physical activity is positively associated with police psychological well-being*(2)*, and so the goal was to co-create an intervention to support police well-being by engaging police workers and their family members in the ‘1TeamActive’ physical activity intervention. Self Determination Theory (SDT*(3)*) has been used to explain the well-being benefits of physical activity*(4) *and was used to theoretically underpin and develop ‘1TeamActive’.* | See Results and logic model (Figure 3) | _____________ |
|  | **WHAT** |  |  |
| **3.** | *Materials:*  *Police force working groups received three support packages to guide the delivery of 1TeamActive in their respective police forces. The support packages included the following:*   - *Support Package 1 – Launch instructions*   - *Action timeline*   - *Communications plan*   - *Advertising media (recruitment documents e.g., poster, screensaver, further information flyer)*   - *Pre-intervention evaluation survey and participant information sheet for participants* - *Support Package 2 – Event planning information for organizers*   - *Event day guidance and equipment requirements*   - *Suggested event layout*   - *Event day schedule*   - *Attendance certificate for participants* - *Support Package 3 – Participant information*   - *Event day information (where to go, what to bring etc)*   - *Event day schedule*   - *Kids activity book*   - *Advice for physical activity instructors*   *Participants completed the pre-intervention survey asking demographic information (age, gender, who they would attend 1TeamActive with) and the quantitative outcome measures. In advance of the event day, participants received the documents in Support Package 3. During the 1TeamActive event day, participants received a short presentation focusing on the benefits of physical activity and nutrition.*  *A Facebook group was created for participants in each police force to access online exercise videos in case they missed a 1TeamActive physical activity session. The Facebook group was also used for participants to connect and share recipes following the nutrition presentation.* | See outcomes measures in 4 (this document). See event day schedule (Supplement File 4). All other materials are available from First Author on request | Materials were available to police forces via the 1TeamActive website (in password protected sections)  <https://www.teampolice.uk/1teamactive/> |
| **4.** | *Procedures:*  *The 1TeamActive Intervention Schedule involved the following:*   - *Registration: Police force workers registered for 1TeamActive by completing a pre-intervention survey, accessed via their in-force advertising and recruitment;* - *Participants were screened by researchers according to eligibility criteria. Eligible participants needed to meet at least one of the following:*   - *Low physical activity – engaged in less than the World Health Organisation (WHO) recommended guidelines for physical activity (measured by the International Physical Activity Questionnaire – Short Form; IPAQ-SF,*(5)*);*   - *Low mental well-being – reported below normative well-being (measured by the Warwick-Edinburgh Mental Well-being Scale (WEMWBS*(6)*);*   - *Participate as part of a family group (e.g., attend 1TeamActive with their spouse and/or children);* - *Eligibe participants were invited onto the 1TeamActive intervention and invited to attend the 1TeamActive event day. At the event days, participants met each other, physical activity instructors, received a presentation on the benefits of physical activity and nutrition, and tried a range of fun physical activities (e.g., Boxercise, active play, hula hooping);* - *At the end of the event day, participants selected the physical activity they wanted to do for the 12-week intervention period. Participants who did not attend the event day made their selection via email;* - *Participants were offered two physical activity sessions per week. Times and locations of each session were arranged between the physical activity instructors and the participants. Retention was monitored over the 12-week intervention period by physical activity instructors;* - *At the end of the intervention period, participants were asked to complete the post-intervention survey (outcome measures only), and whether they would be willing to also participate in an evaluation interview.*   ***Outcomes.***  ***Physical activity.*** *Physical activity was measured using the IPAQ-SF*(5)*. The IPAQ has demonstrated high repeatability, concurrent and criterion validity*(7)*. Over seven questions participants provide the number of days, hours and minutes per day that they have completed various types of physical activity in the last seven days. Answers are converted into total metabolic equivalent (MET) minutes in line with previous research*(7) *and WHO guidelines*(8)*.*  ***Psychological well-being.*** *The WEMWBS*(6) *was used to measure well-being. Tennent et al.*(6) *demonstrated test-retest reliability and internal consistency for this measure, in which positively worded items are used to measure hedonic and eudemonic well-being. Over 14 questions, participants are asked to indicate their experience over the past week by circling a number between one (none of the time) and five (all of the time). For example: “I’ve been feeling optimistic about the future”.*  ***Motivation to participate in physical activity (self-determination).*** *The Psychological Need Satisfaction in Exercise Scale (PNSES*(9)*) was used to measure the three basic psychological needs from SDT across three subscales. High internal reliability has been reported*(10)*. Participants respond on a scale of one (false) to five (true) as to whether they agreed with statements relating to their competence, autonomy or relatedness, for example: “I feel like I get along well with other people who I interact with while we exercise together” was used to measure relatedness.*  ***Active Lives Survey.*** *The Sport England Active Lives Survey*(11) *was used. Only two variables (Anxiety and Self-efficacy/goals) were included in the RM-MANOVA analysis as the other variables in the Active Lives Survey were not continuous variables:*  ***Anxiety.*** *A single item question assessed anxiety. Participants respond on a scale of one (not at all anxious) to ten (completely anxious) to the question: “Overall, how anxious did you feel yesterday?”*  ***Self-efficacy / goals.*** *A single item question assessed self-efficacy/goals. Participants respond on a scale of one (strongly disagree) to five (strongly agree) to what extent they agree to the question: “I can achieve most of the goals I set myself?”*  ***Work productivity.*** *To measure any limitations to work-based productivity, Lerner et al’s*(12) *Work Limitations Questionnaire (WLQ) measure was used. High reliability and construct validity have been reported*(12)*, and the measure has been used in police populations to represent the percentage of productivity lost at work due to health*(13)*. Over eight questions, participants respond on a scale of one (difficult all of the time – 100%) to five (difficult none of the time – 0%) as to how much of the time their physical or emotional health made it difficult for them at work. For example: “In the past 2 weeks, how much of the time did your physical or emotional health make it difficult for you to handle the workload?”* | See participant flow (Figure 2)  See also Results, interview schedule (Supplement File 3) and event day schedule (Supplement File 4). | _____________ |
|  | **WHO PROVIDED** |  |  |
| **5.** | - *TeamPolice – two senior representatives from TeamPolice supported the delivery of the 1TeamActive intervention. They recruited police forces and physical activity instructors onto the intervention via Sport England Active Partnerships, ‘Bark’, a professional hire service, Google search and word of mouth. TeamPolice also set up the support packages for police forces, event day information and coordinated matching participants with their chosen physical activity and instructors;* - *Police force working groups – police force working groups recruited participants within their police forces, supported the delivery of the event days, and liaised between participants, their line managers and TeamPolice to support participants’ continued attendance of 1TeamActive. Police force working group members delivered 1TeamActive as part of their normal work roles;* - *Two academic researchers from Cardiff Metropolitan University (PhDs) supported the delivery of the event days and completed evaluation of the 1TeamActive intervention;* - *Physical activity instructors – led physical activity sessions at the event days and throughout the 12-week intervention. Physical activity instructors had DBS checks, First Aid training and coach/instructor qualifications relevant to their specific physical activity. They were reimbursed for their time;* - *A former professional athlete and coach with a Sport and Exercise Science degree attended the event days to deliver the physical activity and nutrition presentation and was reimbursed for their time.* | See Participants | _____________ |
|  | **HOW** |  |  |
| **6.** | *The event days were delivered face-to-face, to one police force at a time. Following the event day, physical activity sessions were delivered for 12 weeks to participants face-to-face. Participants completed two physical activity sessions per week in groups (all participants with the same physical activity instructors as each other were grouped together). Online exercise videos were also made available via Facebook groups for participants to engage in should they miss a session.* | _____________ | _____________ |
|  | **WHERE** |  |  |
| **7.** | *1TeamActive event days took place in various locations around the UK within each police force area. These comprised a mixture of Police Headquarters, police-owned sports grounds and sports centers. Some sports centers provided their premises for free (recognizing 1TeamActive as a community event), while others required payment. Physical activity sessions over the 12 weeks took place in sport centers, sports halls and gyms – various locations where the physical activity instructors had access as part of their normal role.* | _____________ | _____________ |
|  | **WHEN and HOW MUCH** |  |  |
| **8.** | *The event day was delivered once for each police force. Over the 12 weeks, participants completed two physical activity sessions (approximately one hour each) per week with their physical activity instructors.* | _____________ | _____________ |
|  | **TAILORING** | _____________ | _____________ |
| **9.** | *The same recruitment methods were suggested to all police forces (criterion-based and key informant sampling via Occupational Health Services, health and well-being teams, line managers and police sports clubs; opportunity sampling via poster recruitment, use of screensavers, social media and newsletters). Police forces tailored the advertising methods used as best suited their police force context. For example, Force A were effective in using criterion-based and key informant sampling, because there were two police officers in their working group who were unsuccessful in promotion boards and were looking for development opportunities – this motivated them to recruit as many participants as possible to 1TeamActive and make it a success. Whereas Force D was the smallest police force and found that posters and 1TeamActive screensavers were effective in their context as it ensured that all their workforce saw the intervention information.*  *1TeamActive was also tailored by each police force in terms of the physical activities provided, as these depended on which physical activity providers were available in the local areas. Force C covered the largest police force area; therefore it was not feasible for all participants to travel to attend sessions with any of the physical activity instructors. In this police force, the participants with the longest travel time were allocated gym passes so that they could go to a gym instead of 1TeamActive physical activity sessions. The option for gym passes was not available to all police forces.* |  |  |
|  | **MODIFICATIONS** |  |  |
| **10.^ǂ^** | *None.* | _____________ | _____________ |
|  | **HOW WELL** |  |  |
| **11.** | *Physical activity instructors took registers at each of their 1TeamActive sessions to monitor participant adherence. If any participants were struggling to attend the 1TeamActive sessions then police force working groups and/or TeamPolice would liaise with participants to understand if there was a barrier to their attendance that could be removed. For example, should police shifts be preventing attendance, then police force working groups could liaise with participants’ line managers to facilitate shift swapping. Or, TeamPolice could arrange for participants to swap physical activity instructors.* | _____________ | _____________ |
| **12.^ǂ^** | *Actual:*   - *Attendance data was not completed by all physical activity instructors* - *Attendance data for 58% of the participants was provided (N=59), and, like other authors, we therefore suggest that this data cannot be used to determine ‘how well’ the intervention was received*(14)*.* | _____________ | _____________ |

** **Authors** - use N/A if an item is not applicable for the intervention being described. **Reviewers** – use ‘?’ if information about the element is not reported/not sufficiently reported.

† If the information is not provided in the primary paper, give details of where this information is available. This may include locations such as a published protocol or other published papers (provide citation details) or a website (provide the URL).

ǂ If completing the TIDieR checklist for a protocol, these items are not relevant to the protocol and cannot be described until the study is complete.

* We strongly recommend using this checklist in conjunction with the TIDieR guide (see *BMJ* 2014;348:g1687) which contains an explanation and elaboration for each item.

* The focus of TIDieR is on reporting details of the intervention elements (and where relevant, comparison elements) of a study. Other elements and methodological features of studies are covered by other reporting statements and checklists and have not been duplicated as part of the TIDieR checklist. When a **randomised trial** is being reported, the TIDieR checklist should be used in conjunction with the CONSORT statement (see [www.consort-statement.org](http://www.consort-statement.org)) as an extension of **Item 5 of the CONSORT 2010 Statement.** When a **clinical trial** **protocol** is being reported, the TIDieR checklist should be used in conjunction with the SPIRIT statement as an extension of **Item 11 of the SPIRIT 2013 Statement** (see [www.spirit-statement.org](http://www.spirit-statement.org)). For alternate study designs, TIDieR can be used in conjunction with the appropriate checklist for that study design (see [www.equator-network.org](http://www.equator-network.org)).

**References**

1. Sharp ML, Solomon N, Harrison V, Gribble R, Cramm H, Pike G, et al. The mental health and well-being of spouses, partners and children of emergency responders: A systematic review. PLoS One [Internet]. 2022;17(6 June):1–23. Available from: http://dx.doi.org/10.1371/journal.pone.0269659

2. Oliver H, Thomas O, Neil R, Moll T, Copeland RJ. Stress and psychological well-being in British police force officers and staff. Curr Psychol. 2022;42:29291–304.

3. Ryan RM, Deci EL. Self-determination theory and the faciliation of intrinsic motivation, social development and well-being. Am Psychol. 2000;55(1):68–78.

4. Häusser JA, Mojzisch A. The physical activity-mediated Demand–Control (pamDC) model: Linking work characteristics, leisure time physical activity, and well-being. Work Stress [Internet]. 2017;31(3):209–32. Available from: http://dx.doi.org/10.1080/02678373.2017.1303759

5. Booth M. Assessment of physical activity: an international perspective. Res Q Exerc Sport [Internet]. 2000;71(2 Suppl):S114-20. Available from: http://www.ncbi.nlm.nih.gov/pubmed/10925833

6. Tennant R, Hiller L, Fishwick R, Platt S, Joseph S, Weich S, et al. The Warwick-Edinburgh mental well-being scale (WEMWBS): Development and UK validation. Health Qual Life Outcomes. 2007;5:1–13.

7. Craig CL, Marshall AL, Sjöström M, Bauman AE, Booth ML, Ainsworth BE, et al. International physical activity questionnaire: 12-Country reliability and validity. Med Sci Sports Exerc. 2003;35(8):1381–95.

8. Bull FC, Al-Ansari SS, Biddle S, Borodulin K, Buman MP, Cardon G, et al. World Health Organization 2020 guidelines on physical activity and sedentary behaviour. Br J Sports Med. 2020;54(24):1451–62.

9. Wilson PM, Rogers WT, Rodgers WM, Wild TC. The psychological need satisfaction in exercise scale. J Sport Exerc Psychol. 2006;28(3):231–51.

10. Duda JL, Williams GC, Ntoumanis N, Daley A, Eves FF, Mutrie N, et al. Effects of a standard provision versus an autonomy supportive exercise referral programme on physical activity, quality of life and well-being indicators: A cluster randomised controlled trial. Int J Behav Nutr Phys Act. 2014;11(1).

11. Sport England. Active Lives Survey [Internet]. [cited 2025 Apr 25]. Available from: https://www.sportengland.org/research-and-data/data/active-lives?section=measures

12. Lerner D, Amick BC, Rogers WH, Malspeis S, Bungay K, Cynn D. The Work Limitations Questionnaire. Med Care [Internet]. 2001;39(1):72–85. Available from: http://www.ncbi.nlm.nih.gov/pubmed/11176545

13. Fox J, Desai MM, Britten K, Lucas G, Luneau R, Rosenthal MS. Mental-health conditions, barriers to care, and productivity loss among officers in an urban police department. Conn Med. 2012;76(9):525–31.

14. Cotterill S, Knowles S, Martindale AM, Elvey R, Howard S, Coupe N, et al. Getting messier with TIDieR: Embracing context and complexity in intervention reporting. BMC Med Res Methodol. 2018;18(1):1–10.
