# Supplementary material for: Using the double diamond framework to co‐create and evaluate ‘1TeamActive’: A physical activity and well‐being intervention for police workers and their families
Source: Appl Psychol Health Well Being. 2026 Jul 31;18(4):e70197. doi: 10.1111/aphw.70197 (PMC13428052; doi:10.1111/aphw.70197)
Supplement: Supplementary file 3 — Data S3. 1TeamActive interview schedule. [file APHW-18-0-s004.docx]

1TeamActive interview schedule

| **Section** | **Questions** | **Tot time** |
| --- | --- | --- |
| Introduction | - Hi and thankyou - Explain the purpose of the interview - Explain recording, data storage and transcription processes - Take consent if not completed form already - Reassure – no right or wrong, please be honest - Any questions before we begin? | 5 mins |
| Ice breaker | - Tell me about how you came to be involved in 1TeamActive? - What did you want to get out of the intervention? - How did this differ for other family members? | 8 mins |
| Overall experience | - Overall, how would you describe your experience of 1TeamActive? - What impact, if any, do you think the intervention has had on you personally?   - Physically / mentally / socially? - Has there been any impact on you as a family?   - Do you have any examples of how you supported each other? | 15 mins |
| Event day | - What was your experience at the event day like?   - Can you tell me a bit more about what you enjoyed/didn’t enjoy?   - Could anything have been done differently at the event day, or leading up to the event day?     - If didn’t attend - what barriers were there to attending? What could have helped you to attend?     - PROMPTS: day content, location, schedule…   - What did you think about the presentation?     - If didn’t attend - what did you think about the information you received instead (about instructor sessions)? | 20 mins |
| Instructor sessions | - What did you think about your instructor, and the activities you were doing?   - What did / didn’t you like?   - How much choice did you have? - How would you describe your attendance at the sessions?   - Did this change over the 12 weeks?   - Can you tell me a little more about why you found it hard/easy to attend?   - Was there anything specific that prevented taking part / made it easier to take part? - Did you get to know other people during the intervention?   - Were people supportive, how?   - How could engagement with these parts be encouraged? - What did you and your family gain from 1TeamActive?   - Could you have got more from it? How? | 30 mins |
| Roll out | - Could anything have been done differently in the intervention? - How would you improve it? - Do you think running this project across police forces nationally would work?   - Why / why not?   - How do you think it could run sustainably in your police force? | 35 mins |
| Closing | - Since 1TeamActive ended, have you been able to continue being physically active as a family?   - How did the 1TeamActive intervention help? / How could the intervention have helped, or what could it have done to help you continue being physically active?   - Have their been any other impacts on your lifestyle or family habits? - Is there anything we have not discussed?   - Do you have any questions for me? - Thank you for speaking to me, and for participating | 40 mins |

For children

- What did you think of 1TeamActive?
  - What did you like / not like?
- Tell me about the activities that you did.
  - What was your favourite / least favourite?
  - Did you try anything new?
  - Is there anything new that you learnt?
- What was it like doing the activities with your family?
  - How did you feel during the activities?
- What activities would you like to continue doing?
