# Supplementary material for: Using the double diamond framework to co‐create and evaluate ‘1TeamActive’: A physical activity and well‐being intervention for police workers and their families
Source: Appl Psychol Health Well Being. 2026 Jul 31;18(4):e70197. doi: 10.1111/aphw.70197 (PMC13428052; doi:10.1111/aphw.70197)
Supplement: Supplementary file 4 — Data S4. Supporting Information. [file APHW-18-0-s001.pdf]

1TeamActive

Live Event Day Schedule - 4 activity stations

09.00	Event staff arrival **(1TA) (F)**
09.00	Staff/intro/briefing **(1TA) (F)**
09.15	Event day set up **(1TA) (F)**
**10.45**	Participants arrival and registration **(1TA)**
**11.15**	Welcome inc' safety details/toilets etc **(F)**
**11.20**	Fun warm up **(F)**
**11.40**	Fun relays **(F)**
**12.00**	Activities [10 min rotation] [x2]
**12.30**	**Lunch**<br>Benefits of activity and nutrition presentation [during lunch]
**13.40**	Fun warm up **(F)**
**13.50**	Activity [10 min] [x1]
**14.05**	Activity [10 min] x 1
**14.20**	Fun cool down **(F)**
**14.30**	Farewell message and signpost participants to instructors & hand out certificates. Departure. **(1TA) (F)**
14.30	Event take down [Pack Up] **(1TA) (F)**
15.00	Force feedback/Debrief **(1TA) (F)**
15.15	All persons to leave site
