# Supplementary material for: Using the double diamond framework to co‐create and evaluate ‘1TeamActive’: A physical activity and well‐being intervention for police workers and their families
Source: Appl Psychol Health Well Being. 2026 Jul 31;18(4):e70197. doi: 10.1111/aphw.70197 (PMC13428052; doi:10.1111/aphw.70197)
Supplement: Supplementary file 5 — Data S5. Specific missing measure/scale data points by participant. [file APHW-18-0-s005.docx]

Supplementary File 5. Specific missing measure/scale data points by participant

| Participant code | Missing measure/scale |
| --- | --- |
| AJR0B | IPAC (pre) |
| JCC0N | PNSES-relatedness (pre) |
| JJC0B | Sport England Active Lives Survey (Goals) (post) |
| DXF0B | Sport England Active Lives Survey (Goals) (post) |
| ARB0S | WLQ (post) |
| JDS0H | WLQ (post) |
| AKS0K | WLQ (post) |
| MME2D | WEMBS (post), Sport England Active Lives Survey (Goals) (post), WLQ (post) |
